# Authentication of Chinese vintage liquors using bomb-pulse ^14^C

**DOI:** 10.1038/srep38381

**Published:** 2016-12-06

**Authors:** Peng Cheng, Weijian Zhou, G. S. Burr, Yunchong Fu, Yukun Fan, Shugang Wu

**Affiliations:** 1 State Key Laboratory of Loess and Quaternary Geology, Institute of Earth Environment, CAS, Xi’an, 710061, China; 2 Xi’an AMS Center and Province Key Laboratory, Xi’an, 710061, China; 3 Beijing Normal University, Beijing, 100875, China

## Abstract

The older a bottle of Chinese vintage liquor is, the higher the price it commands. Driven by the potential for higher profits, some newly-founded distilleries openly sell liquor whose storage ages are exaggerated. In China, the market for vintage liquor has become fraught with uncertainty and a pressing need has arisen to establish an effective method to authenticate the age of vintage liquors. A radiocarbon (^14^C) dating method is described here that can verify cellar-stored years of Chinese liquors distilled within the last fifty years. Two different flavored Chinese liquors produced in “the golden triangular region” in the Upper Yangtze River region in southwest China, with known cellar-stored years, were analyzed to benchmark the technique. Strong flavored liquors are found to be consistent with local atmospheric Δ^14^C values. A small offset of 2–3 years between predicted vintage years of soy-sauce flavored liquors and strong flavored liquors is found to be associated with the fermentation cycle of certain varieties. The technique can measure cellar-stored years of a wide range of liquors including those with fundamentally different aromas. This demonstrates the strength of our method in identifying suspect Chinese vintage liquors.

Chinese liquor is one of the world’s oldest distilled alcoholic beverages, and it is typically obtained with the use of *Daqu* fermentation starters^1^. According to historical chronicles, liquor-making in China started 2,000 to 3,000 years ago, about 1,000 years earlier than in western countries^2^. Chinese liquors are typically transparent, colorless or light yellow, with a broad range of aromatic flavors, sweet in taste, and often with very high alcohol contents (typically 40–55 vol.%). Since there is no uniform standard procedure to make Chinese liquor and different raw materials are used in different regions, the aromatic profiles of Chinese liquors are quite different^3^. On the basis of aromatic characteristics, Chinese liquors can be classified into five categories: (1) strong, (2) light, (3) soy sauce, (4) sweet honey, and (5) miscellaneous flavored^4^.

As a traditional distillate, most Chinese liquors are produced from a variety of grains including sorghum, wheat, rice and corn. After several months, or even years of fermentation with daqu, the liquor is distilled with steam. The raw distillates are unsuitable for consumption and are typically aged in cellars until they develop a balanced aroma^5^. While most of the liquors are aged for about 1 year, some of them are aged for more than 3 years. The aged distillate is adjusted to a target ethanol concentration and blended to ensure the quality of the finished product and to maintain brand consistency^4^.

In general, for the same type of Chinese liquor, the longer the aging time the better the taste. Chinese vintage liquor manufacturers cater to consumers’ particular desire for rare liquor with a distinctive aroma and favorable taste. With a growing demand for vintage liquors, a variety of types have emerged, with “3-year”, “5-year”, “30-year” and “50-year” varieties that range from 10 to 5,0000 US dollars per bottle. As one might expect, the older a bottle of liquor is, the higher the price it commands. Driven by the potential for higher profits, some newly-founded distilleries openly sell liquor whose storage ages are exaggerated, and often heavily blended with faints. As a result, the vintage liquor market has become chaotic, infringing on the best interests of the consumer^6^.

Chinese vintage liquors are now being carefully scrutinized by consumers, dampening their otherwise rapid introduction into the market. A number of methods have been reported to verify cellar storage ages, such as atomic absorption analysis^7^ and volatile content^8^. These methods measure the variation of some micro flavor components and liquor color, which are related to their cellar storage ages. However the techniques used are generally restricted to particular liquor varieties. In addition, these techniques cannot be used to determine the proportion of aged liquor into a mixture. The lack of a single and versatile scientific authentication tool contributes to the growing uncertainties surrounding the Chinese vintage liquor market. A reliable identification method is a must to authenticate distilleries, regulate the vintage liquor market and protect the rights and interests of consumers, as well as legitimate producers.

Wines have been dated using radiocarbon at the Oenology Laboratory in Bordeaux, France for some years now^9^. For recent wines, “bomb pulse dating” can be used^10^,^11^,^12^. This method takes advantage of a pulse of anthropogenic ^14^C released into the atmosphere during the years of above-ground nuclear weapons testing. In the Northern Hemisphere, the addition of anthropogenic ^14^C nearly doubled the atmospheric radiocarbon content of the atmosphere by the early 1960s, as compared to pre-bomb levels. This increase halted abruptly in 1963, when a Nuclear Test Ban Treaty was signed, after which atmospheric ^14^C concentrations fell rapidly. This decreasing trend continues today, hastened by the input of fossil fuel-derived CO_2_, which is devoid of radiocarbon^13^,^14^,^15^,^16^,^17^,^18^.

This study utilizes bomb pulse ^14^C dating to determine the year of manufacture for Chinese vintage liquors produced in the “golden triangular region” in the Upper Yangtze River region in southwest China (Fig. 1). The region boasts the largest production and highest quality liquors in China. Water for crops grown in this area come from the Upper Yangtze River. We report results here from liquors with a range of ages and flavors, to test the utility and reliability of the ^14^C dating technique for age authentication of Chinese vintage liquors.

## Results

The ^14^C content of the liquors and tree ring are reported here as Δ^14^C values, which are permil deviations between the sample ^14^C content, as compared to the 1950 AD atmosphere (see below for details).

From the golden triangular region, we sampled a 35-year-old (1980–2014) pine tree (*Larix gmelinii*) at Fenghuang Mountain (27°42′7.74″N, 106°55′20.43″E), Guizhou in 2015, and a 10-year-old (2004–2013) pine tree (*Larix gmelinii*) was sampled from Jinfo Mountain (28°41′9.72″N, 104°43′28.73″E), Sichuan in 2014. These were analyzed to determine a local atmospheric Δ^14^C record in recent years. The results showed that Δ^14^C values varied from 276.5‰ in 1980 to 24.6‰ in 2014. The temporal ^14^C variation can be described as an exponential decline, indistinguishable from the general Northern Hemisphere Zone 3 (NH Zone 3) values^18^ in the atmosphere at the 1σ uncertainty level, apart from 1983 and 1992, which are within 2σ of the NH Zone 3 curve. In the same period. The Δ^14^C value of both regions are consistent with each other at the 1σ uncertainty level, despite of some years with a 3–4‰ variation. The results shown in Supplementary Table S1.

Two of the best-selling types of Chinese liquors (strong and soy-sauce flavor), produced in “the golden triangular region” of China, were obtained. The onset year of the cellar-stored year of liquors, ranging from 1982 to 2012, is decided by the liquor producer. The Δ^14^C contents of a variety of Chinese vintage liquors with strong and soy-sauce flavors are reported in Supplementary Table S2.

**Figure 2:**
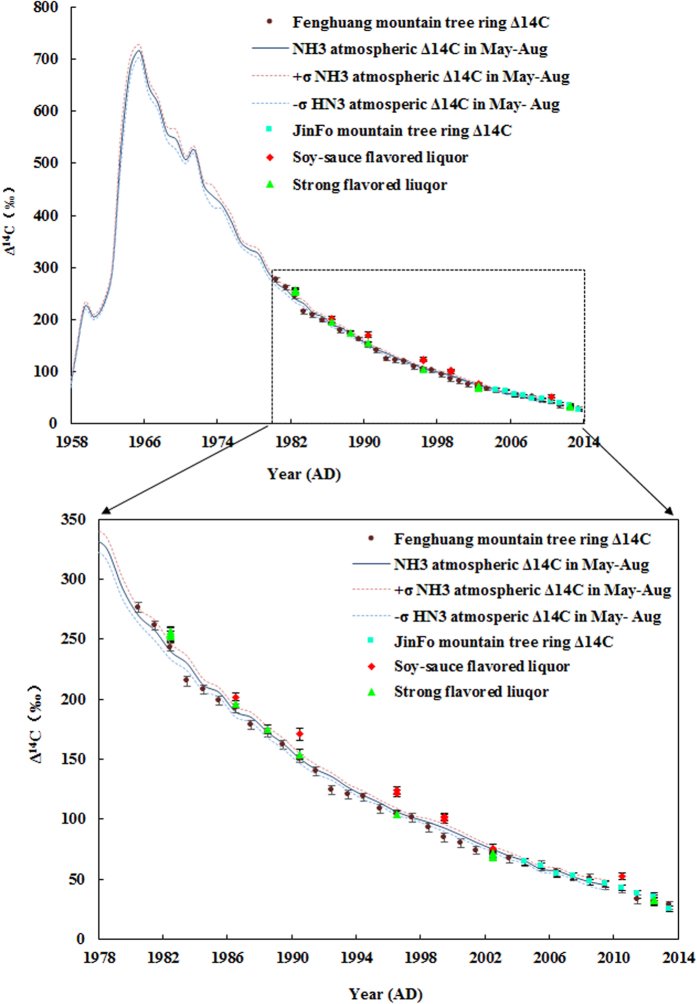
Comparison of Δ^14^C (‰) values in Chinese liquor with local atmospheric Δ^14^C (‰) values from tree rings. Average values of repeat analyses are plotted for the following pairs: (1) XA11931 and XA11932, (2) XA11938 and XA12680, (3) XA11927 and XA11930, (4) XA14584 and XA14587, (5) XA11925 and XA11926.

We observe that the Δ^14^C values of strong flavored liquors are found to be consistent with local atmospheric Δ^14^C values at the 1σ uncertainty level, apart from 1982, whose uncertainties fall within 2σ sigma of the local atmospheric Δ^14^C curve. During the same period, the trend of Δ^14^C of soy-sauce flavored liquors coincides with that of the local atmosphere, however, the Δ^14^C values of the soy-sauce flavored liquors were higher than those of the local atmosphere. For both types of liquors, which were manufactured in the same year, the Δ^14^C values of strong flavored liquors are lower than those of the soy-sauce flavored varieties (Fig. 2), by about 4.3‰ for minimal periods and 19.6‰ for peak periods (see Supplementary Table S2).

**Figure 3:**
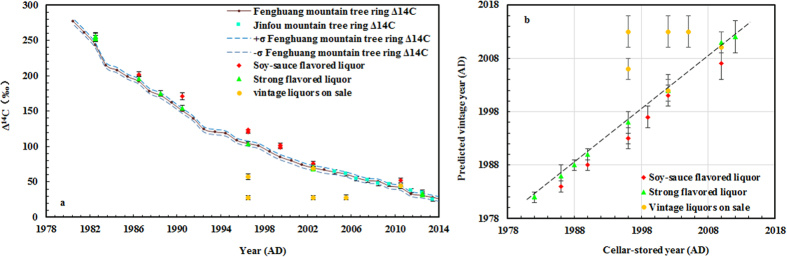
(**a**) Comparison of Δ^14^C (‰) in Chinese liquor with local atmospheric Δ^14^C (‰) from tree rings; (**b**) the relationship of cellar-stored year and predicted vintage year. Dashed line represents a one to one correlation between the cellar-storage year and predicted vintage year. Average values of repeat analyses are plotted for the following pairs: (1) XA11931 and XA11932, (2) XA11938 and XA12680, (3) XA11927 and XA11930, (4) XA14584 and XA14587, (5) XA11925 and XA11926.

We converted our measured liquor Δ^14^C values to predicted vintage year with reference to the local atmospheric Δ^14^C values, and we find that the predicted vintage year of strong flavored liquors are in agreement with their known cellar-stored years. A consistent difference between predicted vintage years of soy-sauce flavored liquors and strong flavored liquors was also observed (Fig. 3b). These differences stem from differences in the time required for fermentation. The results show an age consistent with the time that fermentation materials (grains) were separated from atmospheric carbon exchange (harvest year). Thus, for Chinese vintage liquors, the fermentation cycle needs to be taken into account.

## Discussion

Yibin, Zunyi and Luzhou are all located in the Upper Yangtze River and experience a subtropical monsoon climate. A dense forest coverage creates a favorable ecological environment for grains suitable for high quality liquor production. All of the liquors considered here were produced utilizing the same local grains and under the same local climate. The master liquor-makers in this area have adopted the same basic distillation methodology, incorporating different flavors that are associated with the twenty-four divisions of the Chinese solar year (*jieqi*).

For liquor results, the Δ^14^C values of strong flavored liquors are lower than those of the soy-sauce flavored varieties, about 4.3‰ for minimal periods and 19.6‰ for peak periods (Fig. 2). We believe these differences stem from differences in the time required for fermentation. Soy-sauce flavored liquors are fermented for at least 1 year while strong flavored varieties are fermented for 60–90 days. The fermentation cycle for strong flavored liquors is relatively brief, whereas the fermentation cycle for soy flavored liquors requires more time. That is to say, with the same cellar-stored year, the time required to make a soy-sauce liquor is less than the time required to produce a strong flavored liquor. For liquors with the same flavor, produced in the same areas, Δ^14^C contents vary little from each other, around 1.4‰, as they experience the same fermentation cycle.

We have reconstructed the historic variation of Δ^14^C values in the golden triangular region based on our two tree ring cores. A linear function was fitted to the Δ^14^C data for the period 2004–2013 to determine a 4.3‰ per year rate of decline at Fenghuang Mountain and 3.9‰ per year decline at Jinfo Mountain. These consistent decreasing rates in Δ^14^C from different sites support the use of a local atmospheric Δ^14^C curve for the golden triangular region.

Global atmospheric CO_2_ mixing ratios have rapidly increased over the past several decades. One of the most significant anthropogenic factors is the Suess effect^19^. This is attributed to the emission of carbon dioxide from fossil fuel combustion. The atmospheric Δ^14^C is decreasing exponentially halving approximately every 16 years^20^. Up to 2004, the annual differential in atmospheric Δ^14^C is at most 5‰/year^11^, and will continue to fall. The rate of decrease calculated from a linear data-fit give a value of −9.6‰ per year (R = 0.97 for the period 1980–1999), −4.2‰ per year (R = 0.98 for the period 1999–2009) for tree ring data from Fenghuang Mountain and −9.3‰ per year (R = 0.98 for the period 1980–1999), −4.8‰ per year (R = 0.99 for the period 1999–2009) for NH Zone 3 data^18^. The decreasing trend of the local atmospheric Δ^14^C value is similar with that of NH zone 3^18^. We converted our measured liquor Δ^14^C values to predicted vintage year with reference to the local atmospheric Δ^14^C values (see Supplementary Table S2). During the years 1980 to 1999, the atmospheric Δ^14^C value decreased about 9.3‰ per year. The age difference between predicted vintage years and identified cellar-stored years can be determined for this time span within 1 to 2 years. Since 1999, the Δ^14^C content of the local atmosphere decreased by about 4‰ per year. The age difference between predicted vintage years and cellar-stored years after 1999 can be determined to within 2 to 3 years. Therefore, the cellar-stored years of Chinese liquors for the past 30 years can be resolved with ^14^C dating; however the method becomes less sensitive for liquors produced during the most recent five years as the magnitude of this decreasing trend declines.

We also dated a range of retail vintage liquors, chosen at random, and ranging from 1996 to 2010 years with storage years marked on their labels. Δ^14^C concentrations are listed in Table 1 and plotted in Fig. 3.

Two of the randomly-selected liquor samples (XA11921, XA11925) are consistent with the local atmospheric Δ^14^C curve, but four samples fall well below the curve (Fig. 3a). With the help of the local atmospheric ^14^C data, the predicted vintage year can be determined (see Supplementary Table S2 and Table 1). In a comparison of the cellar-stored years and predicted vintage years (Fig. 3b), most of the data fall along a one to one correlation line, with a negligible offset observed in the case of the strong flavored liquors, due to their relatively shorter fermentation period; and a consistent 2–3 year offset for the soy-sauce flavored liquors, due to their relatively longer fermentation cycle. Much larger age offsets are observed in the four vintage age liquors purchased randomly (Fig. 3b). In these four cases the offsets suggest that the manufacturer’s labeled storage ages are inaccurate.

## Conclusion

We are able to obtain reliable Δ^14^C ages of Chinese vintage liquors using a local atmospheric Δ^14^C curve based on tree rings. In control samples with known cellar-stored years, the data of strong flavored liquors show uniform consistency between known cellar-stored years and predicted vintage years. In a sample of randomly chosen vintage liquors however, we observed significant discrepancies. In contrast to the ^14^C ages of wine, which correspond to the harvest year of the grapes, Chinese liquors require consideration of the fermentation time. For strong flavored liquors this is less than a year, but for soy-sauce flavored liquors, 2–3 years needs to be accounted for to obtain the correct age. The technique described here is widely applicable to vintage liquors, including those with fundamentally different aromas and is an effective means of scrutinizing suspect Chinese vintage liquor products.

## Methods

### Sampling of liquor

Two types of Chinese liquors (strong and soy-sauce flavored) with known ages and produced in the Chishui River Valley, Guizhou, China, were obtained.

Soy-sauce flavored liquors are produced from sorghum and a daqu made of wheat. This type of liquor is full-bodied, mellow, and sweet with a sauce-flavor^21^, referred to as “Mao-scented”, after the best known liquor of this class, Maotai. The traditional production procedure of this type of liquor includes eight stages of fermentation and lasts one year^22^.

Strong flavored liquors are produced from sorghum or other grains and a daqu made of wheat or mixed wheat. These varieties of liquors have pungent pineapple or banana-like fruity aromas^23^. A class of distilled liquor that is sweet in taste, unctuous in texture, and mellow, with a gentle lasting fragrance. Examples of this type of liquor are Lu Zhou and Wuliangye. Their traditional production cycle lasts 60–90 days^24^.

### Sample Pretreatment and measurement

#### Separation of ethanol from liquor by vacuum distillation

A vacuum distillation method was used to extract ethanol from several micro liter liquor samples. This is based on the different saturated vapor pressures of ethanol (100%) (5.7 kPa, at 20 °C) and water (2.34 kPa, at 20 °C). The total vapor pressure of a ~52% (volume concentration) liquor (corresponding to a 25% mole concentration ethanol) will be 3.19 kPa (5.7 kPa × 25% + 2.34 kPa × 75%). Therefore ethanol in the liquor can be extracted under a vacuum at room temperature.

**Figure 4:**
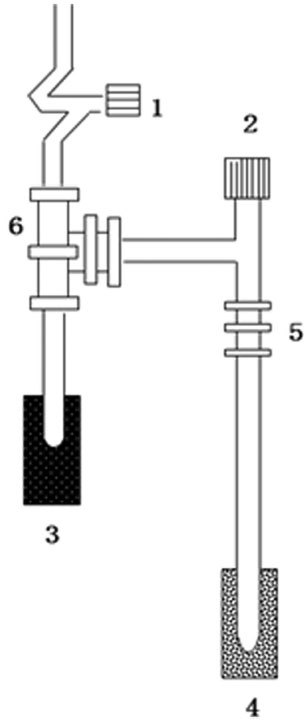
Ethanol extraction device. 1: valve 1; 2: valve 2; 3: liquid nitrogen and alcohol trap; 4: liquid nitrogen trap; 5: vacuum joint; 6: vacuum joint.

The following steps are then followed to extract the ethanol from the liquor (refer to Fig. 4): (1) a 6 mm O.D., 25 mm length quartz tube is filled with 600 mg CuO (baked in advance at 800 °C) to provide the oxygen for the reaction, and connected to vacuum joint 5 and valve 2 is opened to evacuate the copper oxide; (2) when the pressure reaches 1.33 Pa, valve 2 is closed; (3) 8.5 microliters of a 40–55 vol.% liquor sample are inserted into a 9 mm O.D., 10 cm quartz tube, and immediately attached to vacuum valve 6; (4) the liquor sample tube is placed into the ethanol-LN trap (−76 °C) for 2 minutes to freeze the volatile liquor sample, at this temperature atmospheric CO_2_ contained in the quartz tube remains as a gas and can be evacuated without being frozen; (5) valve 1 is opened to evacuate the sample vessel to a pressure of 3.19 kPa and then valve 2 is closed and the ethanol-LN trap is removed to allow the sample to volatilize; (6) valve 2 is opened and the sample is frozen into a 6 mm quartz tube with a liquid nitrogen trap. At this stage the ethanol is contained in a quartz tube with copper oxide. After that, the 6 mm quartz tube is sealed with a torch, removed from the vacuum line and placed into a muffle furnace at 800 °C for 3 hours to oxidize the sample to CO_2_.

#### The extraction of α-cellulose from tree rings

Samples were taken from annual growth rings in pine trees using hollow drills. To obtain sufficient material for AMS analysis, three core samples were taken from each tree and annual growth rings were separated. Each core taken using the hollow drill was 25–30 cm long and 0.5 cm in diameter. The α-cellulose was extracted following a procedure modified from the AAA method^25^: The wood was ground to small pieces and placed into a 100 ml glass tube with 100 ml 1 mol/L HCl solution, pH = 3 at 80 °C for 2 hr, and then NaClO_2_ was added at 1 g for 3 g samples for 2 hr, then filtered, rinsed, and heated in 0.1 M NaOH at 80 °C, for 2 hr. Samples were then filtered, rinsed, and heated again in 0.5 M HCl at 80 °C for 2 hr, then re-filtered, rinsed in distilled water and dried.

The α-cellulose (4 mg) along with cupric oxide was placed in a 6 mm quartz tube and evacuated by a high vacuum system. Once the vacuum level reached 1 × 10^−5^ Torr, the 6 mm quartz tube is sealed with a torch, removed from the vacuum line and placed into a muffle furnace at 800 °C for 3 hours to oxidize the sample to CO_2_.

After combustion, liquid nitrogen and alcohol traps were used to remove H_2_O and other gases. The purified CO_2_ gas was cryogenically transported to a storage vessel for graphitization. The purified CO_2_ was reduced to graphite using a Zn/Fe catalytic reduction^26^,^27^. The graphite samples were then pressed into aluminum holders with a 1 mm internal diameter for ^14^C analysis. Anthracite serves as a background and was measured in order to assess the background in the ^14^C measurements. NBS oxalic acid II (SRM-4990C) was used as a radiocarbon standard.

#### ^14^C dating using an accelerator mass spectrometer

The ^14^C specific activity was calculated from ^14^C/^12^C ratio measurements using the 3 MV AMS at the Xi’an AMS Center, CAS. All samples were measured to achieve better than 3‰ uncertainties for both ^14^C counting statistics and repeat measurement scatter^28^.

The results are reported as Δ^14^C values, which expresses the per mil deviation from the 1950 AD model atmosphere and defined as^29^:


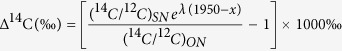


where (^14^C/^12^C)_SN_ is the ^14^C/^12^C ratio of the sample, corrected to δ^13^C = −25‰, (^14^C/^12^C)_*ON*_ is the oxalic acid I standard activity, normalized to 1950 AD, x is the year of growth of the crop used to manufacture the alcohol and λ is the decay constant for radiocarbon (=1/8267 yr^−1^)^29^. The δ^13^C values in Tables S1, S2 and 3 were measured by AMS to correct for isotope fractionation that includes fractionation intrinsic to the samples and fractionation related to the AMS machine.

### Reproducibility Test

Due to the volatile character of liquor, we took splits of the same amount of sample for ^14^C analyses, and made repeat analyses to insure data integrity by way of reproducibility. Hence, we obtained two results, Δ^14^C for each sample (Table 2).

The results shown in Table 2 demonstrate good reproducibility within 1σ sigma errors.

## Additional Information

**How to cite this article**: Cheng, P. *et al*. Authentication of Chinese vintage liquors using bomb-pulse ^14^C. *Sci. Rep.* **6**, 38381; doi: 10.1038/srep38381 (2016).

**Publisher's note:** Springer Nature remains neutral with regard to jurisdictional claims in published maps and institutional affiliations.

## Supplementary Material

Supplementary Information

## Figures and Tables

**Figure 1:**
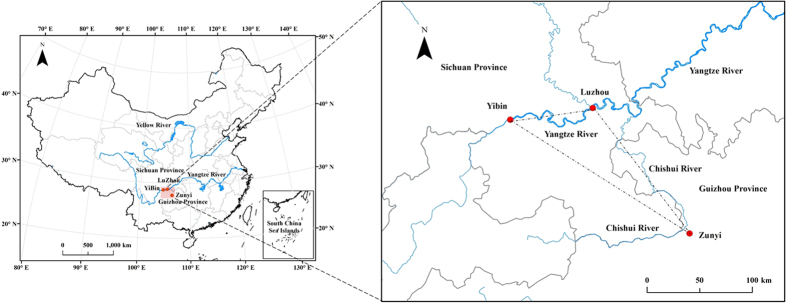
Study area. The smaller map on the left is the map of China, illustrating Guizhou Province and Sichuan Province in southwest China. The map on the right illustrates the study area, located in the convergence region of Guizhou and Sichuan provinces, including Yibin (N28°45′17.01″ E104°38′21.56″) and Luzhou (N28°52′28.94″ E105°26′20.32″) in Sichuan Province, and Zunyi (N27°47′43.76″ E106°23′51.55″) in Guizhou Province, as one of the major liquor-producing regions, referred to as “the golden triangular region”. The liquor and tree ring samples in our study were collected in this region. The map was created by the software ArcGIS 10.2 (Version:10.2.0.3348, www.esri.com).

**Table 1:** The testing results of vintage liquors on sale.

Lab code	Vintage year on the label	δ^13^C (‰)*	Uncertainty	Δ^14^C (‰)	Uncertainty	Predicted vintage year (AD) (1σ intervals)
XA12683	1996	−21.6	0.2	57.2	3.1	2006 ± 2
XA11791	1996	−12.6	0.5	27.4	3.1	2013 ± 3
XA11925	2002	−11.5	0.3	69.5	2.8	2002 ± 2
XA11926	2002	−14.2	0.2	70.2	3.6	2002 ± 2
XA12200	2002	−15.1	0.3	27.8	2.7	2013 ± 3
XA12681	2005	−19.4	0.1	28.1	3.1	2013 ± 3
XA11921	2010	−13.2	0.5	41.4	3.0	2010 ± 3

^*^δ^13^C values were measured in the accelerator mass spectrometer (AMS) for isotope fractionation corrections.

**Table 2:** Reproducibility test of Δ^14^C measurements.

Lab code	Sample code	Δ^14^C (‰)	Uncertainty
XA11931	BJ-1	121.1	2.7
XA11932		123.8	2.9
XA11925	BJ-2	69.5	2.8
XA11926		70.2	3.6
XA12680	BJ-3	71.4	2.9
XA11938		69.0	2.7
XA11930	BJ-4	101.7	2.7
XA11927		101.7	2.8
XA14587	BJ-5	252.5	4.4
XA14584		252.8	4.4

## References

[b1] Zheng X. W., Tabrizi M. R., Nout M. J. R. & Han B. Z. *Daqu-*A Traditional Chinese Liquor Fermentation Starter. J. Inst. Brew. 117, 82–90 (2011).

[b2] Qin H. Z. The Evolution History and Development Trend of Distillate Liquor in China (Part I). Liquor-making Science and Technology. 5, 24–30 (2000) (in Chinese with English abstract).

[b3] Fan W. L. & Qian M. C. Characterization of Aroma Compounds of Chinese “Wuliangye” and “Jiannanchun”: Liquors by Aroma Extract Dilution Analysis. J. Agric. Food Chem. 54, 2695–2704 (2006A). 16569063 10.1021/jf052635t

[b4] Fan W. L. & Qian M. C. Identification of aroma compounds in Chinese ‘Yanghe Daqu’ liquor by normal phase chromatography fractionation followed by gas chromatography/olfactometry. J. Flavor and Fragrance. 21, 333–342 (2006B).

[b5] Fan W. & Qian M. C. Headspace solid phase microextraction and gas chromatography-olfactometry dilution analysid of yound and aged Chinese “Yanghe Daqu” liquors. J. Agric. Food Chem. 53, 7931–7938 (2005). 16190652 10.1021/jf051011k

[b6] Li J. . Identification and management of the Chinese vintage liquor. China Brewing. 31, 6–9 (2012) (in Chinese with English abstract).

[b7] Yang T., Li G. Y. & Zhuang M. Y. Research on Identification Methods of Chinese “Aged Liquor”. Liquor Making. 35, 33–38 (2008) (in Chinese with English abstract).

[b8] Xu Z. C. Study of Scientific Identification Techniques of the Storage Age of Chinese Liquor—Age Liquor Identification by Volatilization Coefficient. Liquor-making Science and Technology. 1, 96–98 (2009) (in Chinese with English abstract).

[b9] Martiniere P. . Evolution de la radioactivite par le carbone 14 des vins de Gironde. Application a la recherche des mill_esimes, Annales des falsifications, de l’expertise chimique et toxicologique. 72, 263–274 (1979).

[b10] Schonhofer F. ^14^C in Austrian wine and vinegar. Radiocarbon. 34, 768–771 (1992).

[b11] Zoppi U. . Forensic applications of ^14^C bomb-pulse dating. NIMB 223–224, 770–775 (2004).

[b12] Asenstorfer R. E., Jones G., Laurence G. & Zoppi U. Authentication of red wine vintage using bomb-pulse ^14^C in Progress in Authentication of Food and Wine (eds Ebeler S. E. .) 89–99 (American Chemical Society, 2011).

[b13] Levin I. . Radiocarbon in atmospheric carbon dioxide and methane: Global distribution and trends in Radiocarbon after four decades: An interdisciplinary Perspective (eds .) 503–518 (Springer-Verlag, 1992).

[b14] Levin I. & Kromer B. The tropospheric ^14^CO_2_ level in mid latitudes of the Northern Hemisphere (1959–2003). Radiocarbon. 46, 1261–1272 (2004).

[b15] Levin I., Kromer B. & Hammer S. Atmospheric Δ^14^CO_2_ trend in Western European background air from 2000 to 2012. Tellus B. Available at: http://dx.doi.org/10.3402/tellusb.v65i0.20092 (2013).

[b16] Hua Q., Barbetti M., Worbes M., Head J. & Levchenko V. Review of radiocarbon data from the atmosphere and tree ring samples for the period AD 1945–1997. IAWA Journal. 20, 261–283 (1999).

[b17] Hua Q. . Progress in radiocarbon target preparation at the ANTARES AMS centre. Radiocarbon. 43, 275–282 (2001).

[b18] Hua Q., Barbetti M. & Rakowski A. Z. Atmospheric radiocarbon for the period 1950-2010. Radiocarbon. 55, 2059–2072 (2013).

[b19] Suess H. E. Radiocarbon concentration in modern wood. Science 122, 415–417 (1955).13246648

[b20] Hua Q., Barbetti M., Zoppi U., Chapman D. M. & Thomson B. Bomb radiocarbon in tree rings from northern New South Wales, Australia: implications for dendrochronology, atmospheric transport and air-sea exchange of CO_2_. Radiocarbon 45 **(3)**, 431–447 (2003).

[b21] Zhu S. K. . Characterization of flavor compounds in Chinese liquor Moutai by comprehensive two-dimensional gas chromatography/time-of-flight mass spectrometry. Analytica Chimica Acta. 2, 340–348 (2007). 10.1016/j.aca.2007.07.007 17683748

[b22] Xiong Z. H. Research on Three Flavor Type Liquors in China (II) Introduction to Maotai—flavor Liquor. Liquor–Making Science and Technology. 40, 25–30 (2005) (in Chinese with English abstract).

[b23] Han B., Xue Y., Li J. & Deng X. W. Rice functional genomics research in China. Phil Trans R Soc B. 362, 1009 (2007). 17347106 10.1098/rstb.2007.2030 PMC2435567

[b24] Li D. H. Relationship of Production Techniques and Quality of Luzhou-flavour Liquor. Liquor–Making Science and Technology. 5, 28–31(2001) (in Chinese with English abstract).

[b25] Zhou W. J., Zhou M. F. & Head M. J. ^14^C chronology of Bei Zhuang Cun sedimentation sequences since 30,000 years BP. Chinese Science Bulletin 35 **(7)**, 567–72 (1990).

[b26] Slota P. J. J., Jull A. J. T., Linick T. W. & Toolin L. J. Preparation of small samples for ^14^C accelerator targets by catalytic reduction of CO. Radiocarbon 29, 303–306 (1987).

[b27] Jull A. J. T. AMS Radiocarbon Dating. In Encyclopedia of Quaternary Science (Second Edition), ed Elias S. A. (Elsevier, Amsterdam), pp. 316–323 (2013).

[b28] Zhou W. J. . The 3MV multi-element AMS in Xian, China: unique features and preliminary tests. Radiocarbon. 48, 285–293 (2006).

[b29] Stuiver M. & Polach H. A. Discussion: reporting of ^14^C data. Radiocarbon. 19, 355–363 (1977).

