# Supplementary material for: Authentication of Chinese vintage liquors using bomb-pulse 14C
Source: Sci Rep. 2016 Dec 6;6:38381. doi: 10.1038/srep38381 (PMC5138620; doi:10.1038/srep38381)
Supplement: Supplementary Information [file srep38381-s1.pdf]

SUPPLEMENTARY INFORMATION

Authentication of Chinese vintage liquors using bomb-pulse ^{14}C

Peng Cheng[1, 2, *, +], Weijian Zhou[1, 2, 3,*, +], G S Burr[1, 2], Yunchong Fu[1, 2], Yukun Fan[1, 2], Shugang Wu[1, 2]

[1] State Key Laboratory of Loess and Quaternary Geology, Institute of Earth Environment, CAS, Xi'an, 710061, China

[2] Xi'an AMS Center and Province Key Laboratory, Xi'an 710061, China

[3] Beijing Normal University, Beijing 100875, China

[*]Corresponding authors. Peng Cheng (email: chp@ieecas.cn) and Weijian Zhou (email: weijian@loess.llqg.ac.cn)

[+] These authors contributed equally to this work

Supplementary Tables

Table S1：$\Delta^{14}C$ results of tree ring from Fenghuang Mountain and Jinfo Mountain in the golden triangular region

Lab Code	Sample Code	year (AD)	$\delta^{13}C$ (‰)*	uncertainty	$\Delta^{14}C$(‰)	uncertainty
XA12929	FH-1	1980	-26.8	0.5	276.5	3.3
XA12930	FH-2	1981	-25.1	0.5	261.3	3.2
XA12931	FH-3	1982	-24.0	0.5	243.5	3.3
XA12932	FH-4	1983	-26.3	0.5	215.0	3.3
XA12933	FH-5	1984	-26.1	0.5	208.0	3.2
XA12934	FH-6	1985	-26.1	0.5	198.6	3.5
XA12935	FH-7	1986	-26.8	0.5	192.2	3.4
XA12936	FH-8	1987	-26.5	0.5	178.4	3.3
XA12937	FH-9	1988	-27.7	0.5	172.3	3.8
XA12938	FH-10	1989	-26.2	0.5	161.9	3.4
XA12939	FH-11	1990	-27.6	0.5	150.5	3.3
XA12940	FH-12	1991	-27.2	0.5	139.8	4.1
XA12941	FH-13	1992	-25.4	0.5	124.2	3.3
XA12942	FH-14	1993	-26.8	0.5	120.6	3.7
XA12943	FH-15	1994	-26.2	0.5	118.5	3.6
XA12944	FH-16	1995	-26.4	0.5	108.5	3.7
XA12945	FH-17	1996	-26.0	0.5	104.4	3.7
XA12946	FH-18	1997	-26.1	0.5	101.0	3.5
XA12947	FH-19	1998	-27.0	0.5	92.7	3.7
XA12948	FH-20	1999	-25.8	0.5	85.0	3.4
XA12949	FH-21	2000	-26.1	0.5	79.9	3.6
XA12950	FH-22	2001	-26.4	0.5	74.0	3.7
XA12951	FH-23	2002	-24.8	0.5	69.9	3.7
XA12952	FH-24	2003	-26.7	0.5	66.8	3.6
XA12953	FH-25	2004	-26.2	0.5	64.0	3.5
XA12954	FH-26	2005	-26.6	0.5	61.1	3.5
XA12957	FH-27	2006	-27.6	0.5	55.8	3.9
XA12958	FH-28	2007	-27.7	0.5	51.9	3.7
XA12959	FH-29	2008	-26.2	0.5	50.7	3.8
XA12960	FH-30	2009	-26.6	0.5	44.3	3.6
XA12961	FH-31	2010	-27.3	0.5	42.0	3.9
XA12962	FH-32	2011	-28.1	0.5	33.1	3.6
XA12963	FH-33	2012	-27.8	0.5	31.0	3.6
XA12964	FH-34	2013	-27.0	0.5	28.0	4.0
XA12965	FH-35	2014	-27.9	0.5	24.6	4.1
XA9146	JF-1	2004	-27.2	0.3	64.5	2.8

XA9016	JF-2	2005	-21.1	0.4	60.9	2.5
XA9023	JF-3	2006	-19.8	0.6	54.3	2.8
XA9022	JF-4	2007	-22.3	0.6	52.4	2.9
XA9021	JF-5	2008	-23.0	0.4	47.4	2.6
XA9140	JF-6	2009	-24.5	0.4	46.0	2.5
XA9143	JF-7	2010	-26.8	0.3	42.7	2.4
XA9018	JF-8	2011	-24.9	0.8	37.4	3.2
XA9017	JF-9	2012	-26.9	0.6	35.1	3.1
XA9015	JF-10	2013	-22.2	0.5	25.0	2.6

*$\delta^{13}C$ were measured by the accelerator mass spectrometer (AMS) for isotope fractionation correction. FH is Fenghuang Mountain, JF is Jinfo Mountain.

Table S2: A comparison of $\Delta^{14}C$ results of Chinese liquors with different flavors, producing areas and storage years

Lab code	Flavour	Cellar-storage year (AD)	Ethanol Vol. (%)	Producing area	Volume (μL)	$\delta^{13}C$(‰)*	Uncertainty	$\Delta^{14}C$(‰)	Uncertainty	Predicted vintage year (AD) (1σ intervals)
XA14587	strong	1982	52	Guizhou	8.5	-6.2	0.8	252.5	4.4	1982±1
XA14584	strong	1982	52	Guizhou	8.5	-5.4	0.8	252.8	4.4	1982±1
XA14585	strong	1982	52	Guizhou	8.5	-6.2	0.9	256.5	4.2	1982±1
XA14583	strong	1982	52	Guizhou	8.5	-6.6	1.0	254.8	4.8	1982±1
XA11807	strong	1986	52	Sichuan	8.5	-11.7	0.4	195.9	3.4	1986±2
XA11935	soy-sauce	1986	53	Guizhou	8.5	-8.8	0.5	202.0	3.4	1984±1
XA11919	strong	1988	52	Guizhou	8.5	-12.7	0.9	175.0	3.8	1988±1
XA11918	strong	1990	52	Sichuan	8.5	-7.2	0.8	154.2	3.8	1990±1
XA11920	soy-sauce	1990	53	Guizhou	8.5	-11.8	0.4	170.8	5.1	1988±1
XA11928	strong	1996	52	Guizhou	8.5	-8.8	0.3	104.2	2.8	1996±2
XA11932	soy-sauce	1996	52	Guizhou	8.5	-7.4	0.8	123.8	2.9	1993±2
XA11931	soy-sauce	1996	52	Guizhou	8.5	-5.8	0.3	121.1	2.7	1993±2
XA11929	soy-sauce	1999	53	Guizhou	8.5	-8.5	0.3	99.1	2.7	1997±2
XA11927	soy-sauce	1999	53	Guizhou	8.5	-6.9	0.4	101.7	2.8	1997±2
XA11930	soy-sauce	1999	53	Guizhou	8.5	-9.9	0.2	101.7	2.7	1997±2
XA12680	strong	2002	53	Sichuan	8.5	-18.9	0.2	71.4	2.9	2002±2
XA11938	strong	2002	53	Sichuan	8.5	-20.1	0.3	69.0	2.7	2002±2
XA11925	strong	2002	53	Sichuan	8.5	-11.5	0.3	69.5	2.8	2002±2
XA11926	strong	2002	53	Sichuan	8.5	-14.2	0.2	70.2	3.6	2002±2
XA11941	strong	2002	52	Sichuan	8.5	-18.7	0.2	68.8	2.7	2002±2
XA11939	strong	2002	52	Sichuan	8.5	-19.5	0.3	68.1	2.7	2002±3

XA12682	soy-sauce	2002	52	Guizhou	8.5	-19.3	0.1	75.7	3.3	2001±2
XA11921	strong	2010	52	Guizhou	8.5	-13.2	0.5	41.4	3.0	2010±3
XA11804	strong	2010	52	Guizhou	8.5	-15.3	0.8	44.3	3.6	2011±2
XA12199	soy-sauce	2010	53	Sichuan	8.5	-18.6	0.3	52.1	2.8	2007±3
XA11800	strong	2012	52	Guizhou	8.5	-12.5	0.6	33.4	3.1	2012±3
XA11795	strong	2012	52	Sichuan	8.5	-12.0	0.5	31.8	3.2	2012±3

*$\delta^{13}C$ were measured by the accelerator mass spectrometer (AMS) for isotope fractionation correction.
